# Remarks, Gleanings, and Extracts

**Published:** 1874-11

**Authors:** 


### REMARKS, GLEANINGS AND EXTRACTS

BY LOCAL EDITORS.

*Treatment of Putrescent Fever.*—C. B. Hall, M.D., (Canada *Lancet*) says:

In 1864, I called the attention of the profession through the Canada *Medical Journal*, to the treatment of those pythogenic diseases of which typhus fever was the type, supporting the theory, that although these diseases are of the continuous class of fever, yet there is a continual change marked by critical days, and at one stage the putrescent symptoms become more marked, and that the regular tendency was this putrescent change, and not resolution, therefore the only reliable remedies were antiseptics, one of which is sulphurous acid. The better way of administering it, in the early stages, when excessive heat, dry skin, flushed face, and constant thirst, require the action of anti-febrifuge and diaphoretic medicines, such as ipecac, solution of ammonia, or perhaps antimony, is in the form of the sulphites of the alkaline bases. This treatment will mostly prevent the fever assuming the more serious symptoms. But for a patient in the state of the case before us, (often found so from neglect of the former medicines,) "the abdomen tympantic, bowels moving every fifteen minutes, each passage containing more or less blood, pulse 120, weak, respiration 30, tongue red at the edges with brown centre and dry as a chip," the most sure and reliable medicine and the most active of its class, is permanganate of potash. This, if given in about a quarter to two grains, and repeated every three hours, will never take more than forty hours to entirely change these appearances. The marked symptoms, as I have given them in the former paper, which indicate the crisis of the pythogenic changes, are the tongue becoming dry, red or brown, with unmistakeable sorders, parched, cracked, the brown increasing to black, incessant thirst, increased pulse, pain in the head, dimness of vision, contracted pupils, ringing in the ears, sleeplessness, wandering of the mind, muttering, muscular tremors and general agitation, all following in rapid succession. This change may take place in twenty-four hours—seldom longer than three days. Sometimes these more marked indications are preceded by the rose-colored spots or petechiæ, all of which show the tendency to putrescency or waste of tissue. Now this is the stage in which I have found permanganate of potash so valuable, indeed I have never seen a failure for over ten years. I do not confine this treatment to pure cases of typhus exclusively, but when the above symptoms are manifest, whether in typhoid pneumonia, scarlet fever, or that more marked and dreaded puerperal fever. If the case has not run into gangrene proper, I have no doubt of the success of this treatment. If not trespassing too much I would cite one or two cases.

Case I.—E. I., male, æt. 20. October 10, 1864. Fourth day of fever, pulse 88, skin hot, dry tongue coated white, bowels confined, no pain in the head, thirst great, urine scanty, no sediment, ordered soda et pot. tart. ʒij., every four hours. 11th. Bowels moved, fæces dark, fœtid, urine slightly increased, pulse 90; prescribed vini ipecac., liq. am. acet. 13th. Complains of pain in the head, pulse 106, tongue clean, red, increased thirst, urine scanty, no deposit; pot. permang. grs. ij., every four hours; soda et pot. tart. ʒij, at night. 14th. Bowels open, stools offensive, tongue dry, cracked, brown; teeth black; delirium; rose spots on abdomen inclining to purple; continue pot. permang. 15th. Tongue moist, clean; urine free, heavy deposit; bowels open. 16th. Stopped pot. permang., and gave infus. cinchona. Convalescent.

Case II.—T. A., æt. 22, female. September 11, 1874. Full habit, strong and generally healthy, was attacked with pain in the stomach the night before, followed by vomiting and purging incessantly, cholera mixtures were tried in vain, mustard and hot cloths had been applied without relief. I saw her first at 4 P. M., the vomiting and purging continued with every movement. Mucus with tinges of blood,—tongue white and coated heavily; pulse 90, feeble; respiration 20; skin dry and hot; excessive thirst for cold water, which was thrown up almost as soon as swallowed. ℞—Sodæ sulphitis, ʒij.; tr. camph. co.; tr. catechu, aa. ℥ss.; aq. cinnam. ad. ℥iv.—M. 7 P.M. vomiting ceased; bowels checked; less pain. Still mucus and blood; continue medicine every two hours. 12th, 9 A.M., better; tongue clean; bowels move every four hours; no return of vomiting. 13th, entirely well. No tonic required. In the last two months such cases have been common, but scarcely any has failed under the use of the sulphites, or the sulpho-carbolate of sodium; nor could any other results be expected if the theory of putrescency be correct, though the convalescence may be prolonged.

*Ergot in the Treatment of Nervous Diseases.*—Dr. Daniel Kitchen, Assistant Physician to the New York State Lunatic Asylum, makes, in the July number of the *American Journal of Insanity*, an interesting report of the action of ergot in certain nervous affections. He used the fluid extract prepared by Squibb, and the aqueous extract, or ergotine, made by Merck, of Vienna. The dose of the former is from one to two drachms; the latter from six to ten grains. One drachm of the alcoholic extract of Squibb's preparation is equal to about six grains of the ergotine. He also used a few ounces of a solid extract, made by Squibb, which is about equal in strength to imported ergotine. The full physiological effect of ergot will last from one-half to three-quarters of an hour.

"There is probably no condition so annoying to the patient as headache, and certainly it is the most common. In the following forms we have used ergotine with much benefit and comfort to the patient: 1. Headache, depending on plethora or fullness of blood; 2. Headache from anæmia; 3. Headache, depending on changes in brain substance and the membranes; 4. Epileptic headaches; 5. Migraine; 6. Headache, depending on disordered menstruation. The most common form of headache is the first, or that depending on a plethoric condition of the blood-vessels of the brain. Of course, we cannot estimate correctly the amount of pain endured at each sickness, but it depends largely upon the constitutional character and nervous susceptibility of the patient. In plethoric headaches the course is either very short (a few hours at most), or they last for some days. The pain is usually referable to the back of the head, and there is much throbbing of the temporal arteries. In this class of headaches we have used ergotine largely; about one hundred patients have been prescribed for, and in almost every instance relief was given in less than half an honr, and the attack thoroughly cut short.

"In headache from an anæmic condition of the brain the blood-vessels are usually lax, and as a consequence there is a slowness of the circulation. Ergotine contracts the blood-vessels, thereby giving tone to the arterial system; the blood is forced more quickly and regularly through the brain, and of course in greater quantity. Our cases of cerebral anæmia are comparatively few, and experiments are therefore limited; yet, in those cases where we have had an opportunity of using it happy results have followed. In epileptic hadaches and in epilepsy we have used ergot largely. In *petit mal* there are no twitchings, congestions of the face, suffusion of the eyes, and a rush of blood to the head. We have in many of these cases been able to ward off the *grand mal* by large doses of ergotine. We have often combined it with conium, and it seems in this combination to work even more satisfactorily than alone, which is due, we suppose, to the sedative effect of the conium. In migraine, or sick-headache, we have distended blood-vessels pressing on the ophthalmic division of the fifth nerve, thereby causing the pain; and if we accept this theory, then ergotine, by contracting the blood-vessels, will relieve the headache. In headaches depending upon some disordered condition of menstruation we usually have a fullness or congestion of the cerebral vessels; sometimes, however, it may occur from anæmia of the brain. In both forms the use of ergotine is beneficial."

Dr. K. concludes his paper with the following statements: "1. Benefit of combination with bromide of potassium in epilepsy; 2. It is apt to produce cramps and pain in the stomach, which is remedied by combination with conium; 3. In nervous diseases it soothes all renal irritation and catarrh of bladder; 4. It dilates the pupil sufficiently to be noticed; 5. Increases both frequency and tension of the pulse; 6. Has no appreciable effect on the heat of the body; 7. In large doses it produces the same effect as conium, by inducing sleep; 8. Its beneficial action in delirium tremens, after bromide of potassium has failed; 9. It combines readily in form of pill with sulphate of quinine; 10. It is a cerebral sedative; 11. Ergotine possesses an advantage over the alcoholic extract in not producing any pain or cramp in the stomach, and is given in smaller quantity; 12. Ergot is not likely to be adulterated, and we always secure an appreciable effect after its administration."

*The Radical Treatment of Hydrocele by Injection of Carbolic Acid.*—A man came to the hospital suffering from a hydrocele of the vaginal tunic of the testicle on the right side, which he stated first began to trouble him a year previous. Six weeks before his admission it had been tapped, and more than a pint of fluid was drawn off, but it rapidly re-developed, and he accordingly presented himself for radical treatment.

As the ordinary mode of treating hydrocele by injecting tincture of iodide into the sac is sometimes unsuccessful, and at other times is followed by an excessive degree of inflammation, and even by suppuration, it was determined to employ carbolic acid as an irritant, which would, it was believed, excite sufficient inflammatory action, and yet, as it checks the formation of pus when externally applied, would have a tendency to limit the inflammation in the sac within the degree of suppuration.

After the serous fluid, which amounted to a pint, had been drawn off by the trocar, the operator injected into the vaginal tunic two fluidrachms of a solution of carbolic acid in glycerin, in the proportion of one part by weight of crystallized acid to two of the menstruum. He then, by manipulation, brought the fluid in contact with every portion of the serous surface, in order that the approximated sides of the sac might be rendered adherent by lymph thrown out upon the supervention of plastic inflammation. The patient did not experience any pain whatever from the introduction of the fluid, such as is the case when tincture of iodide is injected, and which is severe and extends along the course of the genito-crural nerve. It was thought that this painlessness of the procedure might be due to the fact that carbolic acid is capable of inducing local anæsthesia.

At the end of twenty-four hours the tumor was quite large, but had rather a doughy feel, and there seemed to be more inflammation present than generally exists one day after the usual iodine injection has been used; but the swelling was neither painful to the patient nor very sensitive to pressure.

The remarkable feature of the case is the almost entire absence of pain in this method of treating hydrocele.

Carbolic acid seems theoretically to meet all the requirements of the radical cure of hydrocele; but it will require continued experience to determine the practical value of this new method of treatment.—*Dr. Levis, Penn. Hospital.*

*Chloride of Sodium in Dysentery.*—Dr. Woodruff (*Med. and Surg. Jour.*) says: In September, 1855, I read a single paragraph in Nelson's American *Lancet*, recommending salt and morphia as a remedy in sporadic and epidemic dysentery. Being in the midst of an epidemic at the time, I at once resorted to it, and in every case promptly controlled the bloody and frequent evacuations, and distressing tormina and tenesmus, in from twelve to thirty-six hours. When there are evidences of deranged secretions, I premise the treatment with a dose of calomel, (grs. vj.), and opium, (grs. ss.), followed by castor oil, after the operation of which, I give: ℞. Morphiæ, snl., gr. j. to iss.; sodii chloridum, ℈ j. M. Ft. chart, No. vj. Sig. Give one every four hours. After the violence of the attack is relieved, give at longer intervals. This, continued for from one to three or four days, usually completes the cure.

Should there be periodicity, or other symptoms of miasmatic poisoning, of course quinine is demanded, and should be given. In addition to its well-known styptic effect, I believe it exerts a very beneficial effect upon the hepatic secretion, and assists materially to unload the portal circle. In proof of this, I quote from Professor Justus Liebig, who, in his Animal Chemistry, says: "For the production of bile in the animal body a certain quantity of soda is, in all circumstances, necessary; without the presence of a compound of sodium no bile can be formed.

I have suggested the remedy to a number of neighboring practitioners, and invariably received favorable reports.

Of the effects of the prescription, I only "speak that I do know and testify that I have seen."

*Sleeplessness* (*The British Medical Journal*, December 27, 1873). Dr. Dyce Duckworth directs attention to some causes of insomnia, which, he thinks, are hardly sufficiently recognized or adequately met by the resources of practical medicine. Recent researches have clearly shown that the brain is comparatively anæmic during sleep, and that the blood thus removed from the head is more freely supplied to the viscera and integuments.

The most constant cause, and certainly the most frequent accompaniment, of sleeplessness is an opposite condition, or one of active and increased cerebral circulation. A species of nocturnal dyspepsia, mild in its character, and producing no actual suffering, may sometimes give rise to persistent insomnia. There may be no symptoms beyond dryness of the mouth, burning of the soles of the feet, and heat and throbbing in the head, and these are probably due to a too acid condition of the contents of the stomach and upper part of the small intestines, caused generally by excess in fatty and highly-seasoned food, in fruit, and in various wines.

Sleeplessness may be due to bodily and mental over-exhaustion, which results in an increased flow of blood to the brain, consequent upon vaso-motor paresis. Again, it may be the result of mere habit, as in those cases where there has been a long course of broken rest; it may be caused by persistent odors, by certain effluvia, by the absence of moisture in the air of the sleeping apartment, or by an improper elevation or depression of the head.

The treatment in most of these cases should of course be directed to the removal of the cause; but, when it is found necessary to give drugs, bromide of potassium and chloral hydrate are probably the best, both having been shown to diminish the amount of blood circulating through the brain.

*Report Your Cases.*—Every intelligent member of the profession should feel personally bound to contribute to its advancement. In this direction, the following remarks from Dr. Tilt's address before the Obstetrical Society of London are in point, and should be heeded:

"Every now and then there crop up in everybody's practice 'representative' cases—cases which well illustrate a mode of treatment, or confirm some theory, or show the fallacy of another. These are the cases we want, and there can be no excuse for not recording them; for although many of you are too busy to write papers, all can carefully note down the particulars of a case, and we ought all of us to bring ourselves to feel it as a crime to let a little trouble interfere with the careful recording of an important case. If we (this Society) did nothing in the course of each year but to well sift a considerable number of such cases, and to issue them, stamped, as it were, with the seal of authority, we should be laboring most efficiently toward the intelligent reconstruction of medicine; for its unperfection undeniably depends on the deplorable inaccuracy with which cases are collected, if one can call cases the shreds and tatters of half-ascertained facts that we so often meet with in medical works."—*Boston Med. and Surg. Jour.*—*Dental Cosmos.*

*Intra-Recto Abdominal Exploration* (*The Medical Record,* December 1, 1873).—At a meeting of the New York Academy of Medicine, Dr. Charles Leale detailed a case in which he had raised a patient from a condition of profound narcotism from chloroform-poisoning by manual irritation of the solar plexus, the hand having been introduced through the anus and rectum. This treatment had been suggested by the results obtained in a case of like character from the use of electricity over the region of the solar plexus for the purpose of increasing the force of the respiration.

Dr. Benjamin Howard was not sure but that the same effect might have been produced by striking sharply upon the epigastrium where we can come into close proximity to the solar plexus; he thought that, although admissible, the use of both electricity and internal abdominal exploration for the purpose of irritating the solar plexus of nerves as a means of resuscitating should only be employed as a *dernier resort.*

He was of the opinion that this method of examination promises to be an exceedingly valuable one in the diagnosis of tumors and displacements of the uterus, ovarian tumors, etc., and especially in the case of a stricture at the sigmoid flexure of the colon, where so much more can be learned by the touch than in any other way. It has, however, been followed by a strong tendency to incontinence of fæces, and there are cases recorded in which perforation of the bowel and death have resulted from its employment.

Dr. Peaslee would place a check upon the indiscriminate use of this method of exploration. It is justifiable when a differential diagnosis is to be made between a ibroid cyst of the uterus and an ovarian tumor, or between the latter and a tumor coming from above, as an enlarged liver or kidney.—*Phil. Med. Jour.*

*Fistula in Ano.*—Dr. Hute employs an etheral solution of iodine in fistula in ano, which is more exciting than the tincture. Patients are not obliged to keep their beds. He has had several cures after one injection.

*A Large Appetite.*—A woman suffering from bulimia has recently died in Paris. She averaged six and a half pounds of bread, and half pound of meat, daily. If she fed on bread alone it required nine pounds to completely satisfy her appetite.

*Latest Modification of the Cod-Liver Oil Emulsion.*—Those who like cod-oil in emulsion may be glad of a few hints given in the *Archives of Electrology and Neutrology,* which informs us that the last report of the Utica Asylum contains a formula for an emulsion that has long been in use in that institution, and to which attention was fiist called by Dr. Andrews. The writer says he has experimented considerably with various modifications of the original prescription. The latest formula, and one that suits better than any other, is the following: ℞—Cod-liver oil, ℥iv.; glyconin, ℨix. Glyconin is made by thoroughly triturating glycerine and yolk of egg, equal parts. Add to the glyconin thirty drops of the essential oil of bitter almonds; then add the oil to the glyconin *very slowly,* drop by drop, stirring vigorously all the time. The success of the emulsion depends on the thoroughness with which this task is performed. Then add Jamacia rum, ℥ij.; dilute phosphoric acid, ℥ss to ℥i.

The average dose is one tablespoonful after meals, being regulated mainly by the phosphoric acid.

"The above combination is a most excellent brain and nerve food. If properly prepared it does not separate, keeps for a long time, and is rather agreeable to the taste. If need be, pyro-phosphate of iron can be added, or strychnine, or Fowler's solution. We have used it especially in hysteria and allied affections, and in organic diseases of the nervous system it is also valuable. Consumptives take it in preference to cod-liver oil. As cod-liver oil has a somewhat unpalatable name it is sometimes better, in prescribing for nervous patients, to call this the phosphoric emulsion. The fishy odour cannot be entirely neutralized: but for those who are not familiar with cod-liver oil, neither the odour nor taste of this emulsion, when well made, suggest the presence of the oil."—*The Doctor.*

*Hydrastin in Gonorrhœa.*—As there are a great many varieties of treatment in gonorrhœa, I beg to offer a few remarks respecting its mode of treatment, etc. As far as internal treatment is concerned, I merely give in the first stage a saline aperient, to be continued three times daily for four or five days, together with the following injection: Hydrastin, one drachm; solution of morphia (Magendie's), two drachms: acacia mucilage to four ounces: to be used three times daily. This I have employed when inflammation ran very high, without even the slightest ill-effects, and have used it in every stage of gonorrhœa with the most beneficial results when every other treatment, both internally and locally, had failed, including red scondal oil. But there is one remark I wish to make regarding the use of injections which medical men generally forget, and that is, to tell their patients to micturate previous to its use. Unless this is done, injections in gonorrhœa are useless. Hydrastin is used very much in different parts of the United States, and very successfully. My last patient was a farmer, who has had a gleety discharge for seven months. His medical man had quite wearied him out with injections, etc., all to no purpose. I at once tried the hydrastin, and in two weeks he was quite well. *F. N. Bredin, L. R. C. S. I., in Lancet.*

*Ergot in Headache.*—D. R. Silver, of Sidney, Ohio, states in the *Medical and Surgical Reporter*, that ergot is better for headache than any other single article of the Materia Medica. He recommends it to patients who are subject to the malady thus: ℞. Squibb's fl. ext. ergotæ, gtts. x—xx. for one dose. To be repeated every half hour until relief is obtained, or four or five doses are taken. The primary effect of opium is to produce hyperæmia of the brain. To neutralize this action, Dr. Silver employs with it fluid extract of ergot, and says the combination has a happy effect in cases in which opium alone would be contra-indicated by the fluxion of the brain. He does not regard ergot as a specific for headache, but thinks thousands of people are made miserable once a fortnight or once a month, who, by the use of it, may be made, for the time, comfortable. His experience with ergot in the headache of fevers is limited; but he would expect no good result from it, since the pain is then, probably, produced by the abnormal temperature and *quality* of the blood, instead of being occasioned by fullness of the cranial vessels, as once supposed.

*A New Sign of Pyelitis.*—When an alteration in the character of urine is recognized, it is of importance to know if the lesion which gives origin to it is located in the kidney or its pelvis. Several signs have been given by authors to aid in establishing a diagnosis of pyelitis, the most important of which is the presence in the urine of the epithelial cells which line the pelvis and calices. By means of reagents it can be determined if albumen is present or if the quantity of urea is normal. Acid reaction of the urine, according to Oppolzer, is the best symptoms of pyelitis. M. Pascallucci, impressed with the iusufficiency of these means, thinks he has found in the appearance of the crystals of nitrate of urea a more reliable sign. After an examination of the epithelium, he directs that nitric acid should be added and the precipitate examined with the microscope. If catarrh is limited to the bladder the formation of these crystals is normal, that is, they are present under the form of rhomboidal hexagonal plates, joined like roof-tiles. In pyelitis, however, these plates are irregular, the angles cut, and some have the form of little pencils, brooms and feathers. This sign, if constant, is more important than any that have heretofore been maintained.—*Le Movement Med.*, May 10.

*Chloral as an Anæsthetic during Labor* (*The Lancet*, February 21, 1874).—Dr. W. Playfair has found that chloral has the immense advantage over chloroform, when administered during labor, of not lengthening the strength or intensity of the pains, while at the same time markedly diminishing the suffering resulting from them. It is chiefly applicable at a period when we would not think of administering chloroform—towards the termination of the first stage of labor, before the complete dilatation of the os, and when the sharp grinding pains perhaps produce more suffering and are less easily borne than the more forcing pains of a later stage. He gave the drug at first in fifteen-grain doses, and then in smaller quantity, increasing the intervals between its administration, and thus usually keeps up a full and sufficient effect for hours. It need not at all interfere with the exhibition of chloroform.—*Medical Times, Phila.*

*Waterproof Glue.*—Bichromate of potassa has the property of rendering insoluble, under the influence of light, certain organic bodies, such as gum, glue, glycerin, etc. If a paper covered with gum mixed with bichromate is exposed to light, the coating becomes quite insoluble even in boiling water. This property is utilized in the so-called "carbon" photographic process. Strong glue becomes insoluble more rapidly than gum, and the action takes place slowly even in the dark. A concentrated solution of bichromate is prepared, which is kept in the dark, and a little of which is added to boiled gelatin. Objects glued with this after some time can be washed either with cold or hot water.—*Chemical News.*

*Purifying Water.*—It is not generally known that powdered alum possesses the property of purifying water. A tablespoonful of pulverized alum sprinkled into a hogshead of water (the water stirred at the time) will, after the lapse of a few hours, by precipitating to the bottom the impure particles, so purify it that it will be found to possess all the freshness and clearness of the finest spring water. A pailful containing four gallons may be purified by a single teaspoonful.—*Medical and Surgical Reporter.*

*Lead in Syrups of Iodide of Iron.*—Lead, in the form of iodide, has been detected repeatedly in the syrups of iodide of iron, and has been traced to the iodine employed. Lead is not unfrequently present in iron filings, but iron wire had been used in these cases. Resublime iodine alone should be employed.

PROFESSOR HEINE, of Prague, treats chronic enlargements of the prostate by injections per rectum of ten drops of tincture of iodine with twenty of water. Of eleven cases treated by this plan, good results are reported in all.

*Galvano-Cautery.*—In an excellent series of articles on this subject by M. Onimus, the two following propositions are established:

1. Never permit the platinum wire to remain too long in the tissues; withdraw it after it has cauterized to a certain depth and permit it, by contact with the air, to regain a dull red color. In a word, never let division be effected at a temperature which will not at the same time coagulate all the juices.

2. Diminish, by the ordinary means, the circulation of blood in the parts to be cauterized.—*Movement Médical*, May 24, 1873.

*The Efficiency of Enemata.*—Gustav Simon has succeeded in demonstrating that a stream of water forced into the rectum by means of a syringe may be made to penetrate the entire length of the large intestine, and possibly extend also into the small intestine. His experiments were performed upon two separate patients, each of whom happened to have a fistulous opening in the ascending colon, near its junction with the cæcum.—*Archiv für Klinische Chirurgie;* from *Boston Medical and Surgical Journal.*

*Sulpho-Carbolate of Zinc in Pruritis.*—Mrs. J. G. Brown, M.D., of the Illinois Woman's Hospital, (*Med. Examiner,*) recommends as an effectual remedy for obstinate pruritis of the vulva, a solution of sulpho-carbolate of zinc, 30 grains to the ounce of water. After washing with warm water, the solution is applied and left to dry. The application may be made twice a day, to begin with, and afterwards once a day, or two or three times a week.

*Toothache Anodyne.*—Dr. A. G. Craig (Ghent, Ky.) recommends the following as an excellent mixture, which seldom fails to relieve the sufferer of that excruciating torture: Take sulphuric ether, chloroform, of each, 7 fl. drachms; oil of cloves, 2 fl. drachms; gum-camphor, 3 drachms. Mix. Apply on a little cotton, after cleansing out the cavity of the tooth. Some of the liquid may also be rubbed gently on the gums.—*Druggist Circular.*

*A Strange Suggestion.*—The *St. Louis New Era* makes the following strange suggestion. We hardly think it will be carried into effect. It would be a fatal advertisement for some M.D.'s: "In marriage notices it is usual to give the name of the clergyman who performed the ceremony; and with usual propriety, in obituary notices, the name of the attending physician should be given."—*The Doctor*, Nov. 1, 1873.

*Formula for Summer Catarrh.*—Dr. Hoover, in the *American Medical Journal*, recommends a chlorate of potassa, 60 grains, sulph. morph. 12 grains, to six ounces of water, to be used by the atomizer. He says it will give relief immediately, and effect a complete cure in a few days.

